# Sex differences in solid pseudopapillary neoplasm of the pancreas: A population‐based study

**DOI:** 10.1002/cam4.3180

**Published:** 2020-06-23

**Authors:** Jiali Wu, Yize Mao, Yiquan Jiang, Yunda Song, Ping Yu, Shuxin Sun, Shengping Li

**Affiliations:** ^1^ Department of Pancreatobiliary Surgery Sun Yat‐Sen University Cancer Center State Key Laboratory of Oncology in South China Collaborative Innovation Center for Cancer Medicine Guangzhou China; ^2^ Department of Minimally Invasive Intervention Sun Yat‐Sen University Cancer Center State Key Laboratory of Oncology in South China Collaborative Innovation Center for Cancer Medicine Guangzhou China; ^3^ Department of Anesthesiology Sun Yat‐Sen University Cancer Center State Key Laboratory of Oncology in South China Collaborative Innovation Center for Cancer Medicine Guangzhou China

**Keywords:** hormone, SEER, sex‐related discrepancy, solid pseudopapillary neoplasm of the pancreas

## Abstract

**Objective:**

Solid pseudopapillary neoplasm (SPN) of the pancreas is a rare tumor. This study aims to examine the clinicopathological features and surgical treatments of SPN and compare the clinical behavior and prognosis between men and women with SPN.

**Methods:**

We collected the population data of patients with SPN diagnosed between 2004 and 2017 from the SEER database. The Kaplan‐Meier method was used to analyze overall survival (OS) and disease‐specific survival (DSS), and log‐rank tests were used to evaluate the differences between subgroups. Univariate and multivariate Cox regression analyses were performed to screen out prognostic risk factors of SPN.

**Results:**

A total of 378 patients with SPN were included, with 246 (65.1%) female patients. 1‐, 3‐, and 5‐year overall survival rates were 98.9%, 95.7%, and 93.7%, respectively. Survival analysis revealed that regardless of stage, patients with SPN who underwent surgical interventions still had a significantly better prognosis than those without surgical interventions (*P* < .001). The patients with lymphatic dissection had a significantly better prognosis than those without lymphatic dissection (*P* < .001). Moreover, compared with female patients, male patients had significantly poorer OS and DSS (*P* < .001). Female SPN showed a bimodal age‐frequency distribution with early‐onset incidence at 28 years and late‐onset peak incidence at 62 years, while male SPN presented a unimodal distribution with peak incidence at approximately age 64 years. In female patients, the tumor size in premenopausal females (<65 years old) was significantly larger than that in postmenopausal females (≥65 years old) (*P* < .001). Clinicopathological characteristic profiles were different not only between male SPN and premenopausal female SPN but also between premenopausal and postmenopausal female SPN.

**Conclusion:**

SPN presents indolent behavior and predominantly occurs in young women. Regardless of stage, surgical intervention is recommended. Moreover, our study is the first large enough study to demonstrate sex‐related discrepancies in SPN. Thus, different treatment strategies should be designed for patients of different sexes at different ages and hormone therapy is a promising approach for SPN.

## Graphical abstract

Our study is the first large enough study to emphasize the sex‐related discrepancies in solid pseudopapillary neoplasm (SPN), and to observe that clinicopathological characteristic profiles were more common for male SPN and postmenopausal female SPN. It may provide inspiration for different management strategies for male and female patients and imply the possibility of hormone therapy for SPN.

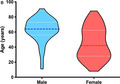

## 1 INTRODUCTION

Solid pseudopapillary neoplasms (SPNs) of the pancreas are uncommon, borderline tumors, accounting for approximately 1%‐2.7% of all pancreatic tumors.^1^, ^2^ Recently, the incidence of SPN has been steadily growing,^3^, ^4^ and with the development and extensive use of imaging, the number of asymptomatic patients with SPN will increase.^5^ It has already attracted increasing attention from clinicians and researchers.

To date, surgical resection remains the optimal treatment for resectable SPN.^6^ Nevertheless, it has been reported that up to 19% of patients with SPN suffer from distant metastasis or localized invasion.^7^ Moreover, recurrence after radical resection can occur in up to 9% of cases,^1^, ^7^ and even after re‐resections, recurrence can still occur.^6^ There is no consensus on treatment for unresectable SPNs, and postoperative recurrence and metastasis, which indicates that other adjuvant treatments should be taken into consideration.

In addition, it has been established that SPN predominantly occurs in women with a women‐to‐men ratio of 10 to 1^7^ and usually affects women of reproductive age.^8^, ^9^ This discrepancy suggests that sex could be a possible epidemiologic risk factor.^10^ Several studies reported that progesterone receptors were present in 79%‐100% of patients with SPN.^11^, ^12^ All of the abovementioned facts suggest that sex hormones may play a role in the pathogenesis of SPN. We hypothesized that there was likely a difference between male and female patients with SPN. However, to our knowledge, a limited number of small case studies comparing men and women with SPN are available, and some controversies remain. Therefore, this study not only aimed to examine the clinicopathological features and the value of surgical treatments for SPN but also to compare the clinical behavior and prognosis between men and women with SPN based on a relatively large cohort study. It may provide inspiration for different management strategies for male and female patients and imply the possibility of hormone therapy for SPN.

## 2 METHODS

### 2.1 Data collection and study cohort

**FIGURE 1:**
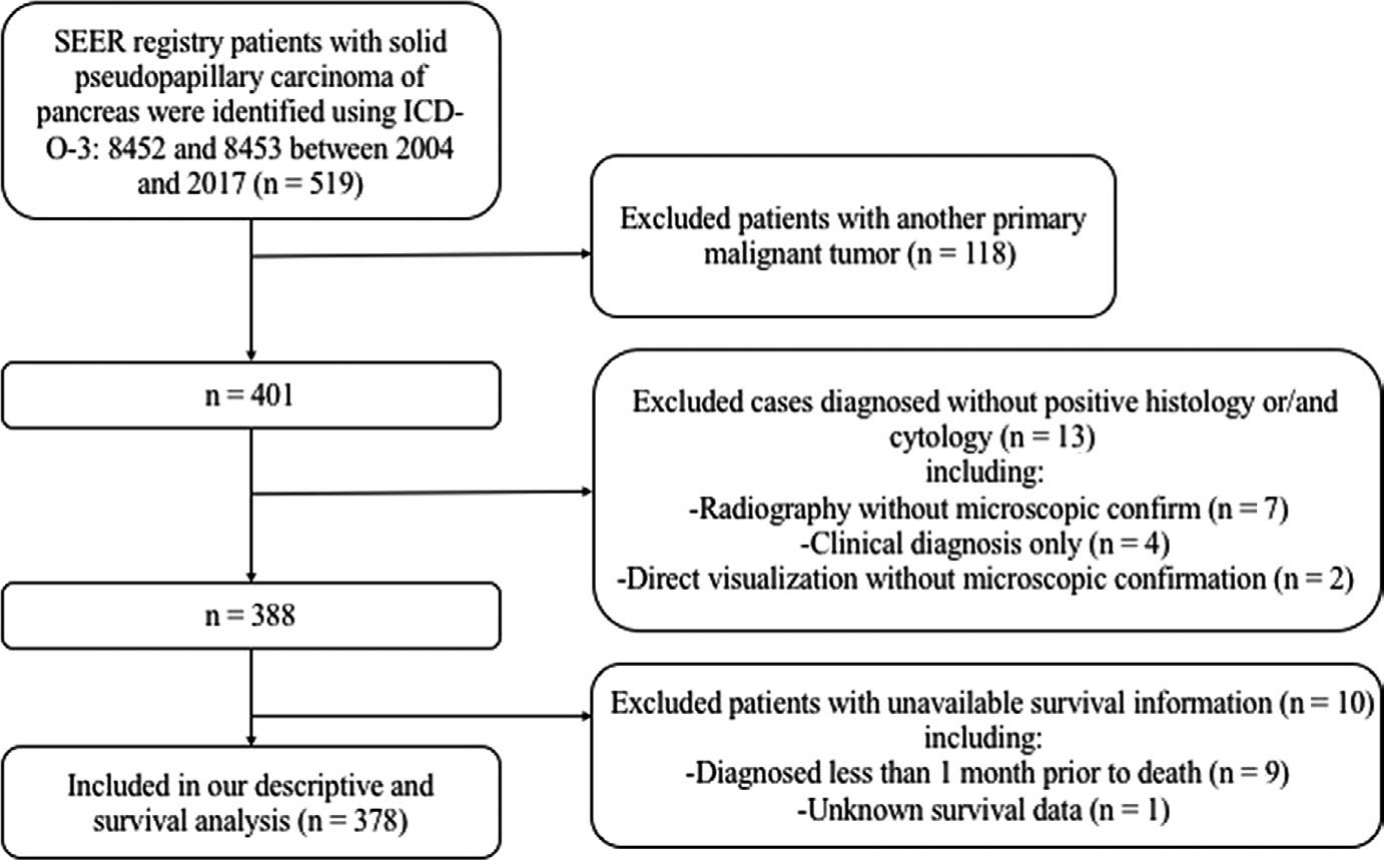
Flow diagram of patients selection in our study from the SEER database 2004‐2017

Data from this study were collected from the the Surveillance, Epidemiology, and End Results (SEER) database of the National Cancer Institute using SEER*Stat 8.3.6 software. We restricted our study to the SEER database (2004‐2017) because detailed Collaborative Stage (CS) data of SPN were not available before 2004. Patients were enrolled in this study if^1^ the ICD Histologic Type ICD‐O‐3 code was 8452 or 8453^2^; SPN was the only primary malignant tumor^3^; patients were diagnosed with positive histology and/or cytology^4^; clinicopathological information for sex, age at diagnosis, year of diagnosis, race, marital status, tumor location, tumor size, T‐classification, number of positive regional nodes, number of examined regional nodes, N‐classification, distant metastasis status, summary stage, surgery types, and radiotherapy or chemotherapy experience was included; and^5^ the vital status and cause of death were known, and patients were diagnosed more than 1 month prior to death. The detailed process of selecting patients is shown in Figure 1.

### 2.2 Study variables

The following variables were obtained from the selected data: sex, age at diagnosis, year of diagnosis, race, marital status, tumor location, tumor size, T‐classification, number of positive regional nodes, number of examined regional nodes, N‐classification, distant metastases status, summary stage, surgery types, radiotherapy or chemotherapy experience, vital status, cause of death, and survival months. According to seer coding manuals (https://seer.cancer.gov/tools/codingmanuals/), all data were precisely decoded. In this study, operation methods are divided into operative treatment (included local excision of tumor, partial pancreatectomy, partial or local pancreatectomy and duodenectomy without distal/partial gastrectomy, Whipple's procedure, extended pancreatoduodenectomy, total pancreatectomy with subtotal gastrectomy or duodenectomy, NanoKnife, and irreversible electroporation) and nonoperative treatment.

### 2.3 Statistical analysis

The patients’ demographic and clinicopathological parameters were summarized by descriptive statistics. Reverse Kaplan‐Meier methods were performed to estimate the median follow‐up time. The chi‐square test or Fisher's exact test was used to compare patient characteristics between the male and female groups. We used Kaplan‐Meier methods to analyze overall survival (OS) and disease‐specific survival (DSS) and log‐rank tests to evaluate the differences between subgroups. Univariate and multivariate Cox regression analyses were used to assess independent prognostic risk factors associated with the OS and DSS of patients with SPN. We presented the results as hazard ratios (HRs) and 95% confidence intervals (CIs). All of the statistical analyses were performed using GraphPad Prism 8 (GraphPad Software) and SPSS Statistics 22.0 (IBM). Differences were considered to be statistically significant when *P* values were less than .05.

## 3 RESULTS

### 3.1 Patient demographic and clinicopathological parameters

In total, 378 patients were identified in this study, and their characteristics in the entire cohort are listed in Table 1. The age of the patients ranged from 8 to 91 years (mean, 50.8 years). There were 277 (73.3%) patients who were white. Fewer than half of the patients were male (34.9%) and unmarried (32.8%). Regarding treatment, most patients never received radiotherapy (90.7%) or chemotherapy (78.8%) treatment. Overall, 330 (87.3%) patients had surgery experience, whereas 48 (12.7%) patients did not undergo surgery. Over half of the SPNs occurred in the head of the pancreas (53.4%), followed by the tail (30.4%) and body of the pancreas (14.3%). The tumor size was more than 40 mm in 191 patients, who accounted for 50.5% of 378 cases. More than half of patients with SPN were diagnosed at the localized stage (57.9%) without positive lymph nodes (84.7%) or distant metastasis (92.3%).

**TABLE 1:** Baseline demographic and clinicopathological characteristics of 378 patients with solid pseudopapillary neoplasm (2004‐2017)

Characteristics	Numbers (n)	Percentage
Age		
≤30	87	23.0
30‐64	177	46.8
≥65	114	30.2
Sex		
Male	132	34.9
Female	246	65.1
Race		
White	277	73.3
American Indian/Alaska Native	3	0.8
Asian or Pacific Islander	47	12.4
Black	47	12.4
Unknown	4	1.1
Marital status at diagnosis		
Married	174	46.0
Single or Unmarried	124	32.8
Divorced or Separated	26	6.9
Widowed	30	7.9
Unknown	24	6.3
Surgical procedures		
0	48	12.7
1	4	1.1
2	166	43.9
3	102	27.0
4	50	13.2
5	8	2.1
Received radiotherapy		
Yes	35	9.3
No	343	90.7
Received chemotherapy		
Yes	80	21.2
No/unknown	298	78.8
Tumor location		
Head of pancreas	202	53.4
Body of pancreas	54	14.3
Tail of pancreas	115	30.4
Unknown	7	1.9
SEER historic stage		
Localized	219	57.9
Regional	128	33.9
Distant	24	6.3
Unstaged	7	1.9
Tumor size (mm)		
Mean ± SD	48.1 ± 33.9	
≤40	181	47.9
>40	191	50.5
Unknown	6	1.6
Lymph nodes positive		
0 LN+	320	84.7
1‐3 LN+	26	6.9
≥4 LN+	16	4.2
Unknown	16	4.2
Distant metastases		
M0	349	92.3
M1	24	6.4
Unknown	5	1.3

Note

Surgical procedures: 0: No surgery of primary site; 1: local excision of tumor; 2: partial pancreatectomy and partial or local pancreatectomy and duodenectomy without distal/partial gastrectomy; 3: Whipple's procedure; 4: Total pancreatectomy and subtotal gastrectomy or duodenectomy and extended pancreatoduodenectomy; and 5: other surgical approaches; LN: Lymph nodes.

### 3.2 Survival analysis and prognostic factors

**FIGURE 2:**
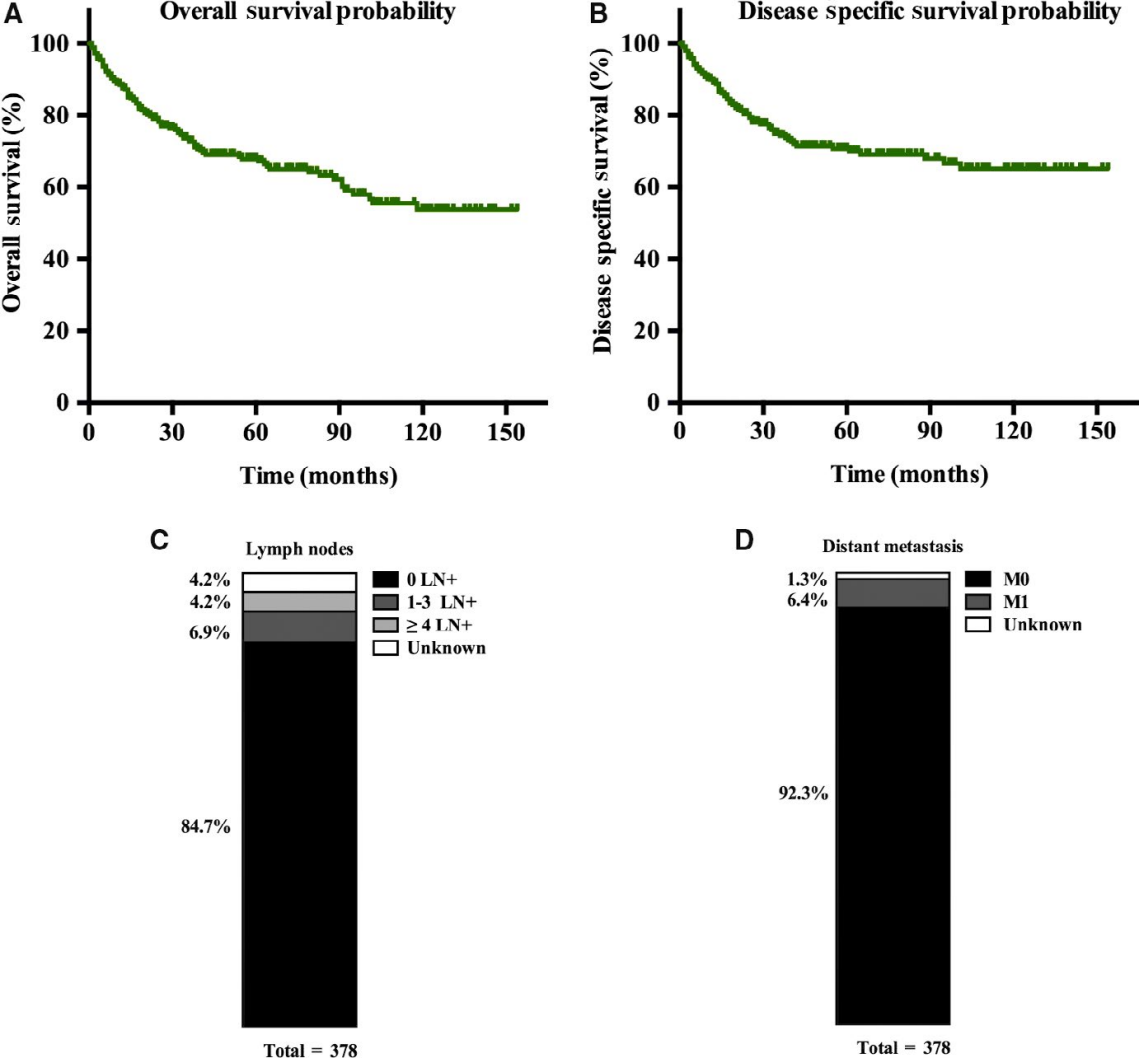
Solid pseudopapillary neoplasm (SPN) exhibited indolent behaviors. A, Survival analysis for overall survival in patients with SPNs; B, Survival analysis for disease‐specific survival in patients with SPNs; C, Lymph nodes metastasis status in SEER cohorts; D, Distant metastasis status in SEER cohorts

In this study, the median follow‐up time was 44 months (range, 1‐154 months). During follow‐up, 103 (27.2%) deaths occurred, of which 80 deaths were attributed to SPN (Table S1). The OS and DSS of patients with SPN are shown in Figure 2A,B. We found that the survival time of patients with SPN was optimistic. The 1‐, 3‐, and 5‐year overall survival rates were 98.9%, 95.7%, and 93.7%, respectively. For DSS, the 1‐, 3‐, and 5‐year survival rates were 99.2%, 96.0%, and 93.2%, respectively. Additionally, we found that only a small proportion of patients with SPN had four or more positive lymph nodes (4.2%) and distant metastasis (6.4%) (Table 1; Figure 2C,D).

**FIGURE 3:**
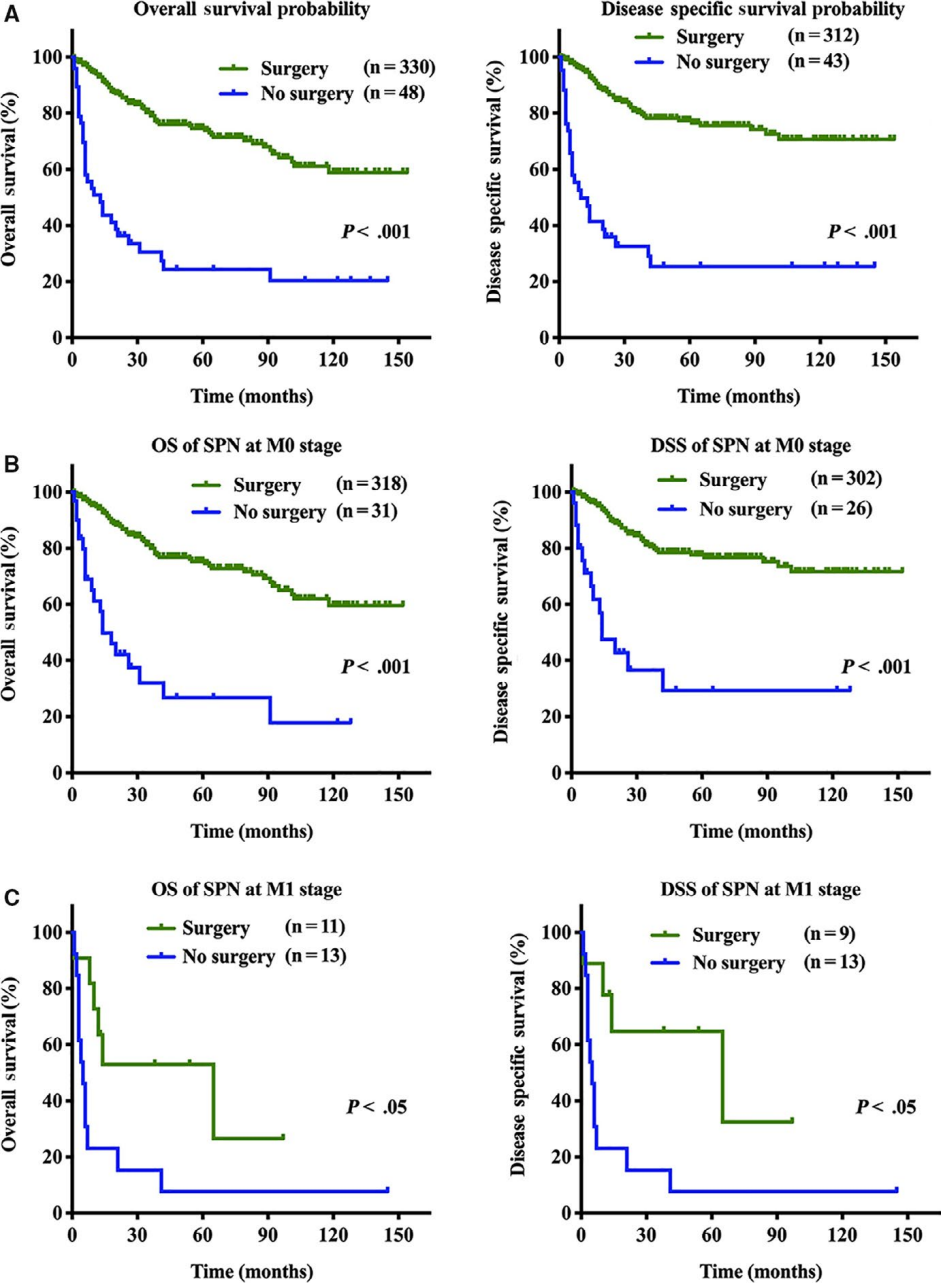
Survival analysis for overall survival and disease‐specific survival in patients with solid pseudopapillary neoplasm based on surgical intervention

**FIGURE 4:**
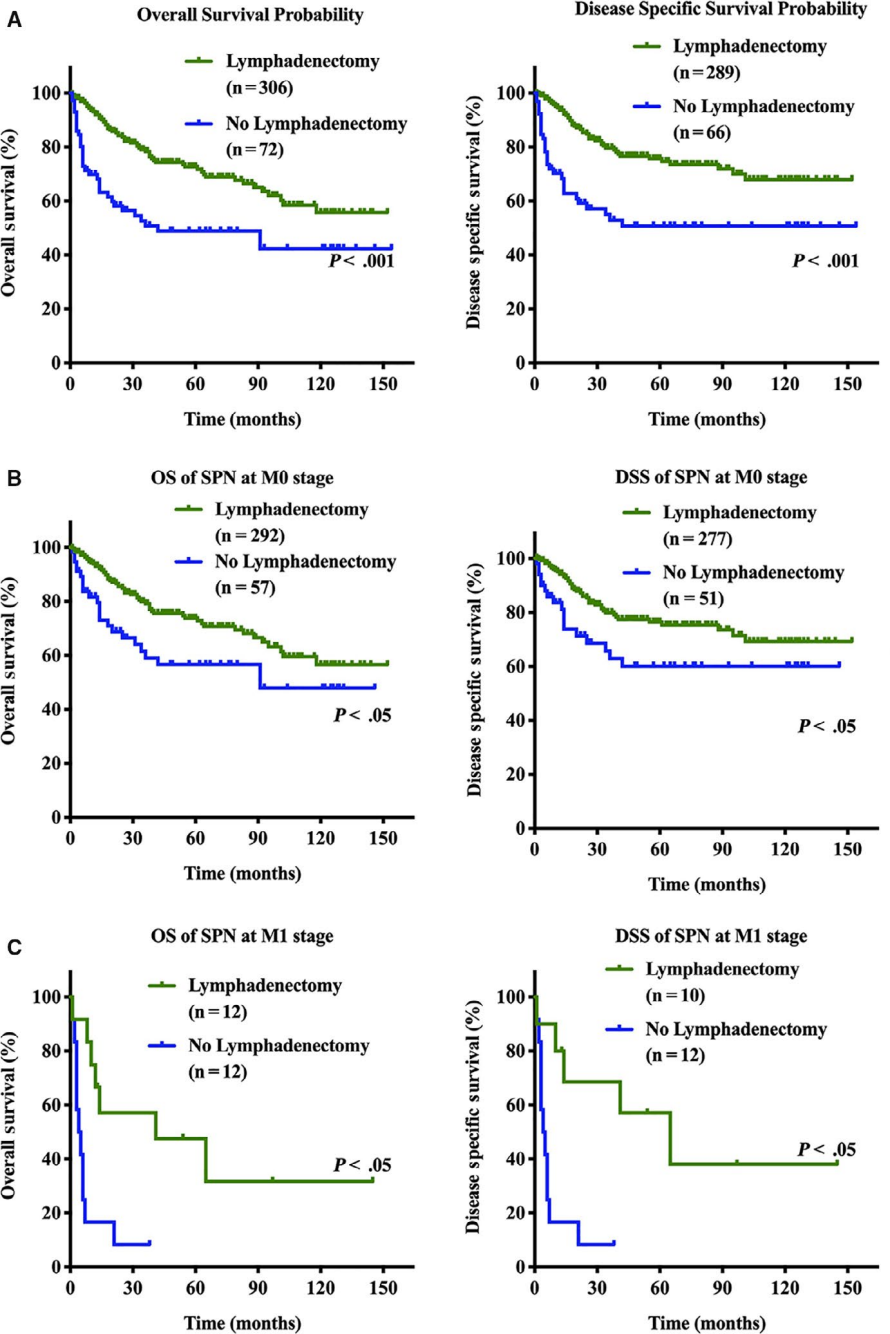
Survival analysis for overall survival and disease‐specific survival in patients with solid pseudopapillary neoplasm based on lymphadenectomy

Next, we used univariate Cox regression to analyze the factors associated with OS and DSS in patients with SPN (Tables S2 and S3). The univariate analysis revealed that age, sex, surgery experience, tumor location, tumor size, lymph node metastasis, and distant metastasis were all significant prognostic factors for OS and DSS of patients with SPN. We found that patients with SPN who underwent surgical interventions had significantly better OS and DSS than those without surgical interventions (*P* < .001; Figure 3). The 1‐ and 3‐year overall survival rates in the surgery group were 98.5% and 97.2%, respectively, compared with 78.8% and 33.5% in the nonsurgery group (*P* < .001; Figure 3A). For DSS, the 1‐ and 3‐year survival rates for patients without surgery interventions were 76.3% and 25.4%, respectively, which were significantly lower than those for patients with surgery interventions (*P* < .001; Figure 3A). Interestingly, we found that regardless of stage, patients still had better survival after surgery than those without surgery (*P* < .001; Figure 3B,C). For lymphadenectomy, patients who received lymphatic dissection had significantly better OS and DSS than those without lymphatic dissection (*P* < .001; Figure 4A‐C).

Prognostic factors of OS and DSS were further assessed by multivariate Cox regression models, which revealed that age (*P* < .001), sex (*P* < .05), surgery experience (*P* < .001), lymph node metastasis status (*P* < .001), and distant metastasis status (*P* < .001) were all independent prognostic factors. However, marital status, tumor size, and tumor location were not found to be significant factors (Tables S2 and S3).

### 3.3 Sex pattern

**FIGURE 5:**
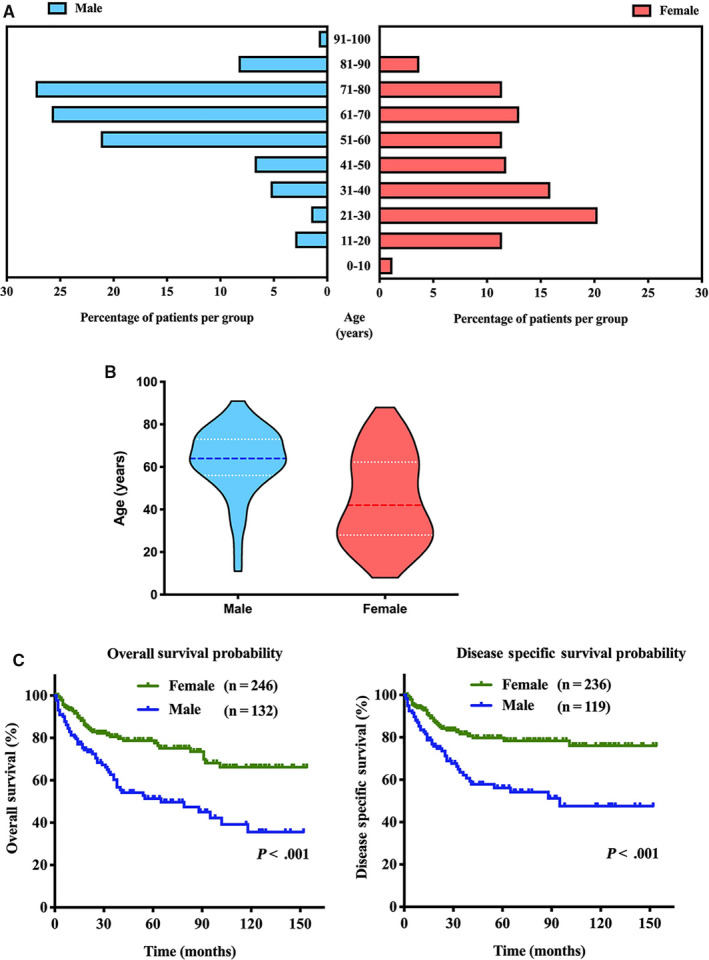
Solid pseudopapillary neoplasm (SPN) presented gender‐related discrepancies. A, Age‐gender distribution of male SPN and female SPN; B, Age‐frequency distribution of male SPN and female SPN; C, Survival analysis for overall survival and disease‐specific survival comparing male and female patients with SPN

Based on sex, we divided the cohort into male and female groups. Their characteristics were compared and are illustrated in Table 2. We observed that female SPN occurred more frequently at the body/tail of the pancreas (54.7%) than male SPN (28.1%). Male patients were less frequently diagnosed at a localized stage (48.5%) than female patients (64.9%). We also found that almost half (49.2%) of male patients were diagnosed at 65 years and older, whereas most (80.1%) female patients were diagnosed younger than 65 years (Table 2 and Figure 5A). As the age‐frequency distribution showed, male SPN demonstrated a unimodal skewness distribution with peak incidence at approximately age 64 years (median age). However, female SPN showed a bimodal distribution with early‐onset and late‐onset peak incidence at 28 (lower quartile age) and 62 years (upper quartile age) (Figure 5B).

**TABLE 2:** Comparison of selected clinicopathological characteristics between male and female with solid pseudopapillary neoplasm

Characteristics	Male		Female		*P*	<65 years female		≥65 years female		*P*
	n	%	n	%		n	%	n	%	
Age										
≤30	6	4.5	81	32.9	**.000** **					
30‐64	61	46.2	116	47.2						
≥65	65	49.2	49	19.9						
Race										
White	103	78.0	174	70.7	.304	134	68.0	40	81.6	.131
Black	13	9.8	34	13.8		31	15.7	3	6.1	
Others/unknown	16	12.1	38	15.4		32	16.2	6	12.2	
Marital status at diagnosis										
Unmarried	28	21.2	96	39.0	**.000** **	90	45.7	6	12.2	**.000** **
Married	78	59.1	96	39.0		76	38.6	20	40.8	
Others/unknown	26	19.7	54	22.0		31	15.7	23	46.9	
Received operation										
No	22	16.7	26	10.6	.090	15	7.6	11	22.4	**.003** *
Yes	110	88.3	220	89.4		182	92.4	38	77.6	
Tumor location										
Head of pancreas	92	71.9	110	45.3	** .000** **	78	39.8	32	68.1	**.000** **
Body/tail of pancreas	36	28.1	133	54.7		118	60.2	15	31.9	
SEER historic stage										
Localized	64	48.5	155	64.9	** .003** *	130	68.4	25	51.0	.065
Reginal	56	42.4	72	30.1		51	26.8	21	42.9	
Distant	12	9.1	12	5.0		9	4.7	3	6.1	
Tumor size (mm)										
≤40	64	49.2	117	48.3	.871	82	42.3	35	72.9	**.000** **
>40	66	508	125	51.7		112	57.7	13	27.1	
Lymph nodes positive										
0 LN+	109	83.2	211	89.4	.089	174	92.1	37	78.7	**.008** *
≥1 LN+	22	16.8	25	10.6		15	7.9	10	21.3	
M										
M0	120	90.9	229	93.1	.122	183	92.9	46	93.9	.713
M1	12	9.1	12	4.9		9	4.6	3	66.1	

Note

The unknown variables were not included.

* *P* < .05.

** *P* < .001.

**FIGURE 6:**
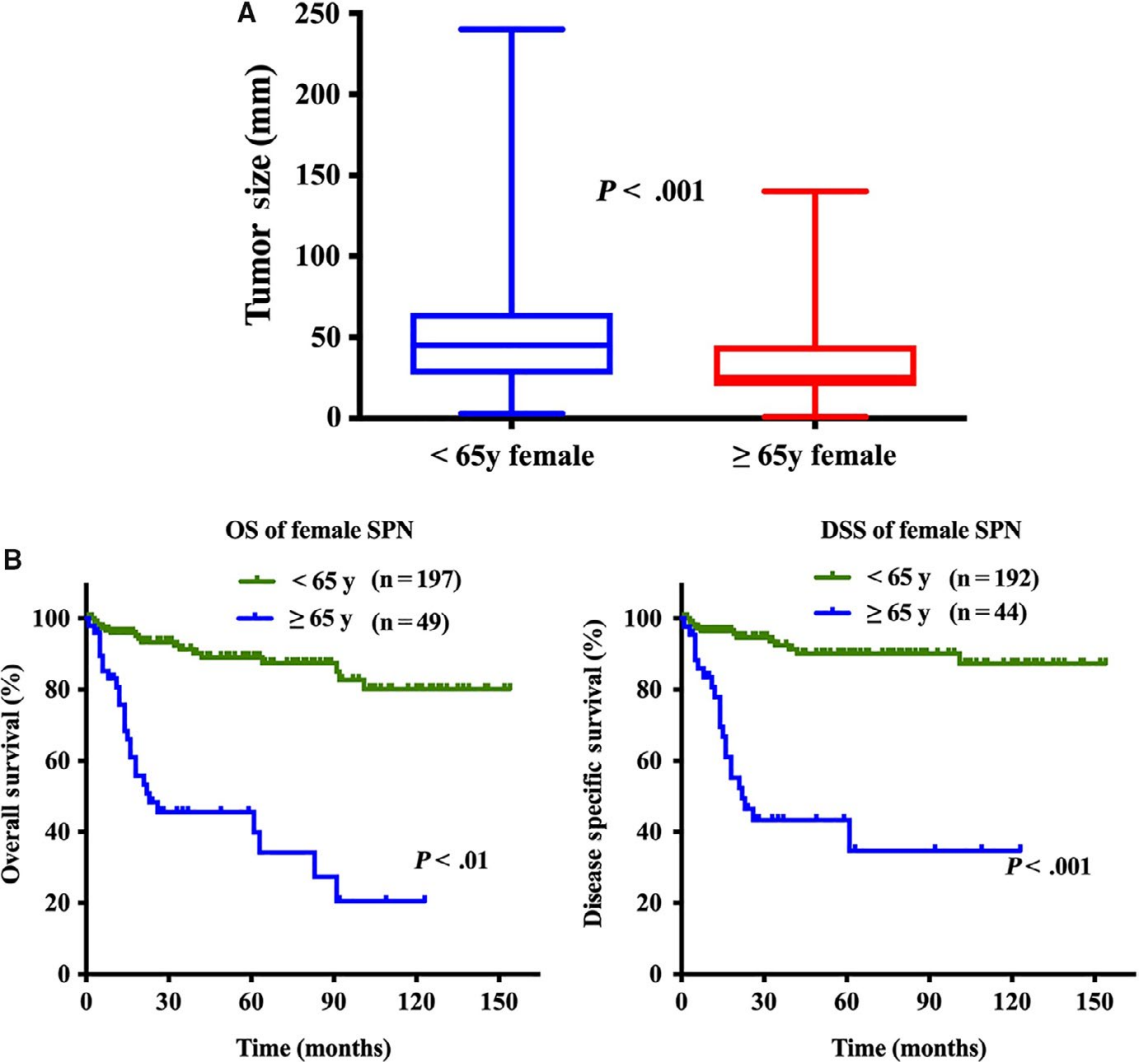
The difference between premenopausal female and postmenopausal female. A, Survival analysis for overall survival and disease‐specific survival comparing premenopausal female and postmenopausal female with solid pseudopapillary neoplasm (SPN); B, Tumor size distribution of SPN in premenopausal female and postmenopausal female

Considering the age of natural menopause and the population characteristics in our cohort, we divided female patients into two groups: younger than 65 years and 65 years and older, representing premenopausal females and postmenopausal females, respectively. Interestingly, we observed that the tumor size in premenopausal females was significantly larger than that in postmenopausal females (Figure 6A).

Survival analysis revealed that male patients had significantly poorer OS and DSS than female patients (*P* < .001, Figure 5C). For female, OS and DSS of postmenopausal patients were significantly poorer than postmenopausal patients (*P* < .001, Figure 6B).

Next, we performed univariate and multivariate analyses based on sex, and we observed that factors affecting survival differed. In male patients, univariate analysis found that factors related to overall survival included age, surgery experience, stage, the number of positive lymph nodes, and distant metastasis status (Table S4). In addition to the above factors, tumor location was found to be a prognostic factor in the female group (Table S4). For DSS, tumor size was significantly associated with prognosis for male SPN (Table S5), but it was not a significant prognostic factor for female SPN. Additionally, tumor site was not a prognostic factor in male patients but was considered a prognostic factor in female patients (Table S5). The multivariate analysis with Cox regression showed that age, surgery performance, and distant metastasis status were independent variables associated with OS and DSS in male patients (Tables 3 and 4). However, distant metastasis status was not found to be an independent prognostic factor for female patients and the number of positive lymph nodes was considered a prognostic factor in female patients (Tables 3 and 4).

**TABLE 3:** Multivariate analysis of factors associated with overall survival for patients with solid pseudopapillary neoplasms

Characteristics	Male			Female		
	*P* value	HR	95% CI	*P* value	HR	95% CI
Age						
<65		Reference			Reference	
≥65	**.015** *	2.159	1.163‐4.009	**.000** **	6.247	2.922‐13.352
Marital status at diagnosis						
Single or unmarried		Reference			Reference	
Married	.504	1.339	0.559‐3.151	.784	1.132	0.467‐2.476
Divorced or separated or widowed	**.009** *	3.990	1.413‐1.267	0.873	1.085	0.101‐2.939
Received surgery						
No		Reference			Reference	
Yes	**.000** **	0.224	0.107‐0.472	**.002** *	0.243	0.098‐0.603
Tumor location	NI					
Head of pancreas					Reference	
Body/tail of pancreas				.266	0.617	0.264‐1.443
Tumor size (mm)				NI		
≤40		Reference				
>40	.921	1.021	0.561‐1.897			
Lymph nodes positive						
0 LN+		Reference			Reference	
1‐3 LN+	.219	0.434	0.114‐1.643	**.000** **	6.034	2.367‐15.377‐
≥4 LN+	**.002** *	4.104	1.660‐10.148	**.000** **	13.404	4.401‐40.823
M						
M0		Reference			Reference	
M1	**.000** **	4.860	2.105‐11.224	.058	3.419	0.960‐12.177

Abbreviation: NI, not included in multivariate survival analysis.

* *P* < .05.

** *P* < .001.

**TABLE 4:** Multivariate analysis of factors associated with disease‐specific survival for patients with solid pseudopapillary neoplasms

Characteristics	Male			Female		
	*P* value	HR	95% CI	*P* value	HR	95% CI
Age						
<65		Reference			Reference	
≥65	**.021** *	2.260	1.129‐4.523	**.000** **	8.698	3.454‐21.907
Marital status at diagnosis						
Single or unmarried		Reference			Reference	
Married	.246	1.804	0.666‐4.886	.903	0.940	0.347‐2.545
Divorced or separated or widowed	**.003** *	6.151	1.890‐20.014	.623	0.740	0.223‐2.454
Received surgery						
No		Reference			Reference	
Yes	**.000** **	0.200	0.085‐0.466	**.001** *	0.162	0.054‐0.487
Tumor location	NI					
Head of pancreas					Reference	
Body/tail of pancreas				.140	0.462	0.165‐1.290
Tumor size (mm)				NI		
≤40		Reference				
>40	.984	0.993	0.487‐2.024			
Lymph nodes positive						
0 LN+		Reference			Reference	
1‐3 LN+	.171	0.361	0.084‐1.555	**.000** **	7.318	2.538‐21.097
≥4 LN+	**.021** *	3.515	1.209‐10.218	**.000** **	16.937	5.111‐56.132
M						
M0		Reference			Reference	
M1	**.000** **	6.573	2.417‐17.877	.057	4.436	0.954‐20.620

Abbreviation: NI, not included in multivariate survival analysis.

* *P* < .05.

** *P* < .001.

## 4 DISCUSSION

In our study, we analyzed the SEER database of 378 patients with SPN and mainly elaborated the following three points:

First, we evaluated the biological behavior of SPNs by analyzing demographic and clinicopathological characteristics. Consistent with conventional views, we observed that SPN exhibited indolent behaviors with a relatively low risk of lymph node metastasis (12.4%) and distant metastasis (4.5%) in the SEER cohort. Most patients with SPN were diagnosed at the localized stage (85.1%). We also found that the SPN showed a favorable prognosis. The 1‐, 3‐, and 5‐year DSS rates were 98.9%, 95.7%, and 93.7%.

Second, we assessed the effect of surgery treatments on patients with SPN. Previous evidence confirmed that surgical resection was an effective approach to treat SPN, and satisfactory outcomes could be obtained by tumor debulking.^13^, ^14^, ^15^ In our study, we demonstrated similar results in that patients with SPN after surgical resection had significantly better OS and DSS than those without surgical resection. Meanwhile, we identified that patients diagnosed at distant stages still had more favorable survival after surgical debulking than those without surgical debulking. However, the validity of lymphadenectomy remains a controversial issue. Some researchers claimed that there was no need to undertake formal lymphadenectomy routinely,^16^, ^17^ whiles other reported that lymphatic dissection is necessary.^14^, ^18^ We found that patients with lymphatic dissection had a significantly better prognosis than those without lymphatic dissection. Thus, surgical resection guarantees the good prognosis, but further studies need to evaluate the effectiveness of lymphatic dissection. Moreover, in view of long‐term survival and low‐grade malignancy, organ‐preserving surgery, such as duodenum‐preserving pancreatic head resection and spleen‐preserving resection, and laparoscopic surgery will be encouraged in specialized pancreatic centers.^19^, ^20^, ^21^

Last but not least, investigating the sex feature of SPNs. In our current study, SPN showed a predilection for females in 65.1% of all cases. It is worth noting the distinctly different patterns of onset between males and females. We revealed that female SPNs showed a bimodal age‐frequency distribution, while male SPNs demonstrated a unimodal skewness distribution. Moreover, we found that female SPN had an early‐onset incidence at 28 years and a late‐onset peak incidence at 62 years, while male SPN had a peak incidence at approximately 64 years of age. Previous evidence implied that sex hormones might participate in the pathogenesis of SPN.^20^, ^22^ Strong immunoreactivity for progesterone has been identified in many studies.^11^, ^12^, ^23^ Case reports showed that SPN grew rapidly during pregnancy,^24^, ^25^, ^26^ and progesterone might act as an oncogenic factor in SPN. For estrogen, Tognarini I et al demonstrated the strong expression of ER in tumor tissue and the proliferative action of estrogen in vitro, offering potential treatment strategies for SPN via selective ER modulators.^27^ Similarly, another study reported that antiestrogen drugs for cases with unresectable liver metastasis resulted in a favorable prognosis.^28^ Therefore, in our study, we presumed that the early onset in women might be attributed to exposure to progesterone and/or estrogen during the reproductive period, while the late onset might be attributed to accumulated lifetime environmental exposure.

The age of natural menopause varies by individual, race, and ethnicity, with a range of 40‐60.^29^, ^30^ Considering the age of natural menopause and population characteristics in our cohort, we divided female patients into two groups: younger than 65 years and 65 years and older, representing premenopausal females and postmenopausal females, respectively. Interestingly, we observed that the tumor size in premenopausal females was significantly larger than that in postmenopausal females. It indicated that female hormones affect the growth of SPNs and that anti‐female hormone agents may be a promising adjuvant. However, the function of female hormones in development has not yet reached consensus and needs further study.

Our study also revealed that male patients had significantly poorer OS and DSS than female patients. This difference may be because male SPNs had a higher percentage of patients older than 65 years than female SPNs. Reported surveys and our analysis consistently identified that older age was an independent prognostic risk factor for SPN.^10^, ^31^ In addition, we found that the clinicopathological characteristic profiles were more similar for male SPN and postmenopausal female SPN. To our knowledge, our study is the first to describe this phenomenon in a male population. Moreover, it could partly explain that despite the larger tumor size in premenopausal females, the OS and DSS of premenopausal females were significantly better than those of postmenopausal females.

There are several limitations in our current population‐based study. First, due to the indolent behavior of SPNs, it was difficult to conduct large prospective studies. Our study is retrospective and may inevitably include biases, which affect the analysis. Second, the rarity of SPN in nature and the lower number of patients in the subgroups based on sex and age results in limited statistical power. Third, the lack of detailed information about chemotherapy and radiation made it difficult to evaluate their effect on SPN. Meanwhile, information on recurrence was not recorded, and factors that contributed to the sex disparities, such as serum levels of estrogen, progestogen, androgen, menstrual status, and reproductive histories, were not available in the SEER database. Therefore, it is difficult to precisely assess the difference between female and male patients with SPN at different ages. To better understand these complicated sex disparities and the role of sex hormones in the development of SPN, further clinical and fundamental research is needed. Despite these limitations, this is the first study that is large enough to demonstrate that male SPN was different from female SPN, which suggests that different treatment strategies should be designed for patients of different sexes at different ages and implies the possibility of hormone therapy.

## 5 CONCLUSION

In conclusion, this population‐based study demonstrated that SPN present indolent behavior and predominantly occurs in young women. Regardless of stage, surgical intervention is recommended. Moreover, our study is the first large enough study to emphasize the sex‐related discrepancies in SPN, and to observe that clinicopathological characteristic profiles were more common for male SPN and postmenopausal female SPN. Thus, different treatment strategies should be designed for patients with different genders at different ages and hormone therapy was a promising approach.

## CONFLICT OF INTEREST

None of the authors have any potential conflict of interest to declare.

## AUTHOR CONTRIBUTIONS

SL, JW, and YM designed the project. JW, YJ, PY, and SS performed the experiments and data extraction. JW, YM, YJ, and YS contributed statistical analysis. JW and YM wrote the manuscript. All authors reviewed the manuscript.

## Supporting information

Supplementary Materials

## Data Availability

All data generated for this study are available from the corresponding author upon request.
