# Supplementary material for: Sex differences in solid pseudopapillary neoplasm of the pancreas: A population‐based study
Source: Cancer Med. 2020 Jun 23;9(16):6030–41. doi: 10.1002/cam4.3180 (PMC7433837; doi:10.1002/cam4.3180)
Supplement: Supplementary file 1 — Supplementary Materials [file CAM4-9-6030-s001.docx]

**Supplementary Tables**

**Table S1. Baseline Survival parameters of 378 Patients with SPNs (2004-2017)**

| Parameters | Total | | Male | | Female | |
| --- | --- | --- | --- | --- | --- | --- |
|  | n | % | n | % | n | % |
| Cause of death |  |  |  |  |  |  |
| Alive | 275 | 72.8 | 74 | 56.1 | 201 | 81.7 |
| Dead of SPN | 80 | 21.1 | 45 | 34.1 | 35 | 14.2 |
| Dead of other cause | 23 | 6.1 | 13 | 9.8 | 10 | 4.1 |
| Median follow-up time (range) | 44(1-154) | | 61(1-152) | | 33(1-154) | |

**Table S2. Univariate and Multivariate Analysis of Factors Associated with Overall Survival for Patients with SPN**

| Characteristics | Univariate analysis | | | Multivariate analysis | | |
| --- | --- | --- | --- | --- | --- | --- |
|  | P value | HR | 95% CI | P value | HR | 95% CI |
| Age |  |  |  |  |  |  |
| < 65 |  | reference |  |  | reference |  |
| ≥ 65 | **<0.001**** | 4.692 | 3.413-7.005 | **<0.001**** | 3.133 | 1.963-5.002 |
| Sex |  |  |  |  |  |  |
| Male |  | reference |  |  | reference |  |
| Female | **<0.001**** | 0.422 | 0.285-0.622 | **0.008*** | 0.545 | 0.349-0.851 |
| Marital status at diagnosis |  |  |  |  |  |  |
| Single or Unmarried |  | reference |  |  | reference |  |
| Married | 0.062 | 1.653 | 0.975-2.804 | 0.677 | 1.133 | 0.630-2.036 |
| Divorced or Separated or Widowed | **<0.001**** | 3.410 | 1.918-6.063 | **0.026*** | 2.043 | 1.090-3.831 |
| Tumor location |  |  |  |  |  |  |
| Head of pancreas |  | reference |  |  | reference |  |
| Body/Tail of pancreas | **<0.001**** | 0.453 | 0.293-0.701 | 0.938 | 0.980 | 0.587-1.635 |
| Received Surgery |  |  |  |  |  |  |
| No |  | reference |  |  | reference |  |
| Yes | **<0.001**** | 0.198 | 0.131  0.301 | **<0.001**** | 0.220 | 0.135-0.357 |
| Tumor size (mm) |  |  |  |  |  |  |
| ≤ 40 |  | reference |  |  | reference |  |
| ＞40 | **0.030*** | 0.646 | 0.436-0.959 | 0.343 | 0.819 | 0.543-1.237 |
| Lymph nodes positive |  |  |  |  |  |  |
| No |  | reference |  |  | reference |  |
| Yes | **<0.001**** | 3.720 | 2.232-5.831 | **<0.001**** | 3.260 | 1.929-5.508 |
| M |  |  |  |  |  |  |
| M0 |  | reference |  |  | reference |  |
| M1 | **<0.001**** | 4.704 | 2.821-7.841 | **<0.001**** | 2.345 | 1.287-4.272 |

^*^P ＜ 0.05, ^**^P ＜ 0.001

**Table S3. Univariate and Multivariate Analysis of Factors Associated with Disease-Specific Survival for Patients with SPN**

| Characteristics | Univariate analysis | | | Multivariate analysis | | |
| --- | --- | --- | --- | --- | --- | --- |
|  | P value | HR | 95% CI | P value | HR | 95% CI |
| Age |  |  |  |  |  |  |
| <65 |  | reference |  |  | reference |  |
| ≥ 65 | **<0.001**** | 4.909 | 3.121-7.723 | **<0.001**** | 3.121 | 1.843-5.284 |
| Sex |  |  |  |  |  |  |
| Male |  | reference |  |  | reference |  |
| Female | **<0.001**** | 0.409 | 0.263-0.637 | **0.026*** | 0.566 | 0.343--0.936 |
| Marital status at diagnosis |  |  |  |  |  |  |
| Single or Unmarried |  | reference |  |  | reference |  |
| Married | 0.128 | 1.575 | 0.878-2.825 | 0.478 | 1.1270 | 0.657-2.453 |
| Divorced or Separated or Widowed | **<0.001** | 3.569 | 1.873-6.800 | **0.019*** | 2.377 | 1.153-4.899 |
| Tumor location |  |  |  |  |  |  |
| Head of pancreas |  | reference |  |  | reference |  |
| Body/Tail of pancreas | **0.001*** | 0.428 | 0.261-0.701 | 0.902 | 0.964 | 0.533-1.742 |
| Received Surgery |  |  |  |  |  |  |
| No |  | reference |  |  | reference |  |
| Yes | **<0.001**** | 0.159 | 0.100-0.252 | **<0.001**** | 0.242 | 0.132-0.445 |
| Tumor size (mm) |  |  |  |  |  |  |
| ≤ 40 |  | reference |  |  | reference |  |
| ＞40 | **0.008*** | 0.539 | 0.340-0.853 | 0.410 | 0.814 | 0.499-1.328 |
| Lymph nodes positive |  |  |  |  |  |  |
| No |  | reference |  |  | reference |  |
| Yes | **<0.001**** | 4.236 | 2.596-6.913 | **<0.001** | 3.397 | 1.928-5.987 |
| M |  |  |  |  |  |  |
| M0 |  | reference |  |  | reference |  |
| M1 | **<0.001**** | 5.433 | 3.134-9.419 | **0.010*** | 2.375 | 1.234-4.568 |

^*^P ＜ 0.05, ^**^P ＜ 0.001

**Table S4. Univariate Analysis of Factors Associated With Overall Survival for Paitents With SPNs**

| Characteristics | Male | | | Female | | |
| --- | --- | --- | --- | --- | --- | --- |
|  | P value | HR | 95% CI | P value | HR | 95% CI |
| Age |  |  |  |  |  |  |
| < 65 |  | reference |  |  | reference |  |
| ≥ 65 | **0.004*** | 2.205 | 1.292-3.763 | **< 0.001**** | 7.844 | 4.299-14.458 |
| Marital status at diagnosis |  |  |  |  |  |  |
| Single or Unmarried |  | reference |  |  | reference |  |
| Married | 0.990 | 1.005 | 0.494-2.045 | 0.146 | 1.810 | 0.813-4.029 |
| Divorced or Separated or Widowed | **0.010*** | 2.927 | 1.286-6.662 | **0.001*** | 3.848 | 1.699-8.716 |
| Received Surgery |  |  |  |  |  |  |
| No |  | reference |  |  | reference |  |
| Yes | **< 0.001**** | 0.180 | 0.100-0.323 | **< 0.001**** | 0.196 | 0.105-0.366 |
| Tumor location |  |  |  |  |  |  |
| Head of pancreas |  | reference |  |  | reference |  |
| Body/tail of pancreas | 0.593 | 0.848 | 0.463-1.553 | **0.002*** | 0.375 | 0.199-0.708 |
| SEER historic stage |  |  |  |  |  |  |
| Localized |  | reference |  |  | reference |  |
| Reginal | 0.146 | 1.539 | 0.861-2.748 | **< 0.001**** | 3.586 | 1.866-6.891 |
| Distant | **< 0.001**** | 7.178 | 3.494-14.747 | **< 0.001**** | 6.048 | 2.364-15.472 |
| Tumor size (mm) |  |  |  |  |  |  |
| ≤ 40 |  | reference |  |  | reference |  |
| > 40 | 0.079 | 0.619 | 0.363-1.057 | 0.152 | 0.649 | 0.359-1.172 |
| Lymph nodes positive |  |  |  |  |  |  |
| 0 LN+ |  | reference |  |  | reference |  |
| 1-3 LN+ | 0.876 | 1.085 | 0.390-3.020 | **< 0.05*** | 4.191 | 1.816-9.671 |
| ≥4 LN+ | **0.003*** | 3.481 | 1.510-8.022 | **< 0.001**** | 12.692 | 4.792-33.612 |
| M |  |  |  |  |  |  |
| M0 |  | reference |  |  | reference |  |
| M1 | **< 0.001**** | 5.889 | 3.5054-11.357 | **0.004*** | 3.515 | 1.485-8.323 |

^*^P ＜ 0.05, ^**^P ＜ 0.001

**Table S5. Univariate Analysis of Factors Associated With Diease-Specific Survival for Paitents With SPNs**

| Characteristics | Male | | | Female | | |
| --- | --- | --- | --- | --- | --- | --- |
|  | P value | HR | 95% CI | P value | HR | 95% CI |
| Age |  |  |  |  |  |  |
| < 65 |  | 1 |  |  | 1 |  |
| ≥ 65 | **0.015*** | 2.104 | 1.156-3.828 | **< 0.001**** | 9.290 | 4.631-18.638 |
| Marital status at diagnosis |  |  |  |  |  |  |
| Single or Unmarried |  | 1 |  |  | 1 |  |
| Married | 0.958 | 1.022 | 0.457-2.285 | 0.232 | 1.688 | 0.715-3.983 |
| Divorced or Separated or Widowed | **0.008*** | 3.391 | 1.371-8.391 | **0.010*** | 3.412 | 1.346-8.649 |
| Received Surgery |  |  |  |  |  |  |
| No |  | 1 |  |  | 1 |  |
| Yes | **< 0.001**** | 0.148 | 0.079-0.280 | **< 0.001**** | 0.161 | 0.080-0.325 |
| Tumor location |  |  |  |  |  |  |
| Head of pancreas |  | 1 |  |  | 1 |  |
| Body/tail of pancreas | 0.669 | 0.864 | 0.443-1.685 | **0.002** | 0.311 | 0.149-0.650 |
| SEER historic stage |  |  |  |  |  |  |
| Localized |  | 1 |  |  | 1 |  |
| Reginal | 0.512 | 1.255 | 0.636-2.475 | **< 0.001**** | 4.242 | 1.999-8.999 |
| Distant | **< 0.001**** | 7.546 | 3.526-16.149 | **< 0.001**** | 7.347 | 2.550-21.168 |
| Tumor size (mm) |  |  |  |  |  |  |
| ≤ 40 |  | 1 |  |  | 1 |  |
| > 40 | **0.031*** | 0.498 | 0.265-0.937 | 0.126 | 0.590 | 0.300-1.160 |
| Lymph nodes positive |  |  |  |  |  |  |
| 0 LN+ |  | 1 |  |  | 1 |  |
| 1-3 LN+ | 0.662 | 1.260 | 0.447-3.556 | **< 0.001**** | 5.390 | 2.131-13.635 |
| ≥4 LN+ | **0.033*** | 2.872 | 1.091-7.561 | **< 0.001**** | 15.586 | 5.710-42.543 |
| M |  |  |  |  |  |  |
| M0 |  | 1 |  |  | 1 |  |
| M1 | **< 0.001**** | 9.512 | 4.394-20.589 | **0.005*** | 3.876 | 1.498-10.024 |

^*^P ＜ 0.05, ^**^P ＜ 0.001
